# Development and Evaluation of a Transportation Planning e-Tool for Older Adults: Co-Design Study

**DOI:** 10.2196/88297

**Published:** 2026-10-23

**Authors:** Sara Bahrampoor Givi, Dany Baillargeon, Sahar Tahir, Bessam Abdulrazak, Mireille Gagnon-Roy, Mélanie Levasseur, Nathalie Delli-Colli, Patrick Boissy, Mélisa Audet, Véronique Provencher

**Affiliations:** 1 Research Center Charles-Le Moyne, Longueuil, QC, Canada; 2 School of Rehabilitation, Faculty of Medicine and Health Sciences, Université de Sherbrooke, 3001, 12e Avenue Nord, Sherbrooke, QC, J1H 5N4, Canada, 1 8198218000; 3 Research Center on Aging, Centre intégré universitaire de santé et de services sociaux de l'Estrie—Centre Hospitalier Universitaire de Sherbrooke, Sherbrooke, QC, Canada; 4 Department of Communication, Université de Sherbrooke, Sherbrooke, QC, Canada; 5 Department of Computer Science, AMI-Lab, Faculty of Science, Université de Sherbrooke, Sherbrooke, QC, Canada; 6 School of Social Work, Université de Sherbrooke, Sherbrooke, QC, Canada; 7 Department of Surgery—Orthopedics, Université de Sherbrooke, Sherbrooke, QC, Canada; 8 Centre collégial d’expertise en gérontologie du Cégep de Drummondville, Drummondville, QC, Canada; 9 Faculty of Letters and Human Sciences, Université de Sherbrooke, Sherbrooke, QC, Canada

**Keywords:** older adults, user experience, participatory design, e-tool, transportation

## Abstract

**Background:**

Older adults often face challenges using digital transportation planning tools to engage in meaningful activities. In Sherbrooke, a Canadian city located in the province of Québec, hilly terrain, bus-based mobility, and varied digital literacy among older adults highlight the need for accessible transportation planning technologies. Despite the growing demand for accessible and inclusive technologies, interfaces are often not tailored to the specific needs and preferences of older adults, making them difficult to use. Addressing this gap is essential to support the mobility and social participation of older adults in their communities.

**Objective:**

This study aimed to enhance older adults’ experience with a transportation planning e-tool—called Mobilainés—by co-designing its interface and evaluating its usability and user experience in line with their needs and preferences.

**Methods:**

Using a participatory design approach, the study encompassed both development and evaluation phases, integrating qualitative and quantitative methods. In the development phase, heuristic evaluation, along with the Mobilaînés mock-up, was performed to identify the percentage of compatibility with age-friendly usability design principles and guidelines. Fourteen co-design workshops with the steering committee and research team informed persona development and core functionalities, while 3 workshops with older adults refined the mock-ups. For the evaluation phase, 2 laboratory usability tests (simulated context) and 1 in-field test (real-world context) were conducted with 5, 7, and 4 older adults, respectively. Data were collected using a combination of objective performance metrics recorded in real time by a computer and subjective user feedback, gathered using the System Usability Scale, the Unified Theory of Acceptance and Use of Technology questionnaires, and focus group discussions. For the in-field testing, user experience data were obtained via the User Experience Questionnaire and semistructured interviews. Quantitative data were analyzed descriptively, and qualitative data were analyzed thematically.

**Results:**

The participatory design and iterative evaluation process identified key design decisions for age-friendly transportation planning, including step-by-step navigation, simplified task flow, clear feedback, visual clarity, and personalization. In the final laboratory usability test, the Mobilaînés e-tool showed 84% compatibility with age-friendly guidelines, a 97% task completion rate, and System Usability Scale and Unified Theory of Acceptance and Use of Technology scores corresponding to very good usability. In-field User Experience Questionnaire results and interviews suggested that participants perceived the e-tool as reliable, easy to use, and accurate, despite the lack of real-time data.

**Conclusions:**

Participatory design and iterative usability testing revealed that older adults’ experience with Mobilainés transportation planning e-tools is improved. These design learnings contributed to improved usability and confidence in real-world travel planning, offering practical guidance for the development of age-friendly digital transportation tools. Future research should explore collaborative and technological strategies to support its adoption and sustained use, particularly among older adults with limited digital literacy.

## Introduction

### Background

Worldwide, the aging population will increase from 900 million (2015) to 2 billion by 2050 [1]. In Canada, it is expected to reach 15.1 million by 2063 [2]. Age-related changes, such as declining vision [3], reduced mobility [4], and cognitive decline, may hinder traffic signs recognition, decrease flexibility, and slow reaction times while driving [5], which can limit older adults’ ability to drive safely and travel independently [6]. Additionally, financial constraints [7], rural living [8], and small social networks [9] further restrict access to transportation options. These challenges increase the risk of sedentary behavior, isolation [10,11], loneliness [11], and anxiety [12]. To enable older adults to maintain their independence and engage in community life—key elements of social participation [13]—it is therefore essential to strengthen older adults’ ability to access and use transportation options that reflect their specific needs, including those involving emerging technologies.

Digital transportation planning tools (websites and apps) are intended to support users in accessing information, selecting transportation options, and organizing their travel. While these tools have the potential to improve mobility, they often pose considerable usability challenges for older adults. Age-related changes, such as decreased visual acuity [14], reduced contrast sensitivity [15], and diminished dexterity [16], can make it difficult for them to identify important interface elements, interpret maps, and accurately interact with touch or clickable components [17]. In addition to these sensory and motor declines [4], cognitive changes associated with aging, such as reduced executive functioning, can impair an individual’s ability to navigate complex transportation systems or coordinate multistep travel plans [6]. Digital literacy [18] also remains a key barrier; limited familiarity with technology can restrict access to real-time transportation updates or adaptive features within e-tools [19]. Differences in numerical comprehension and screen navigation further emphasize the need for design solutions that reflect the diverse abilities of this population [20].

Beyond usability considerations, digital transportation planning tools may influence older adults’ mobility and well-being through psychological and cognitive mechanisms. Accessible and intuitive interfaces can reduce cognitive demands, helping users process information more efficiently and make travel decisions with less effort [21]. This may enhance perceived control over travel and strengthen mobility-related self-efficacy, understood as confidence in one’s ability to plan and complete trips, particularly because older adults’ transition to public transit use involves acquiring travel-planning skills and confidence through experience [22]. Together, greater perceived control and self-efficacy may encourage more frequent and independent use of transportation options, supporting social participation and well-being. Conversely, poorly designed interfaces may increase cognitive burden, reduce confidence, and discourage engagement with transportation systems [23].

Addressing such specific needs of older adults is vital for creating inclusive tools that enhance their user experience and support independent mobility. A participatory design approach is especially valuable for ensuring that these needs are fully integrated into the development and evaluation of transportation planning interfaces. Participatory design, sometimes known as cocreation or co-design [24], is described as a collaborative process in which various participants engage in shared exploration, understanding, and learning through ongoing reflection and action [25]. Among other benefits, participatory design contributes to a better user experience, as it is conceived with the end users in mind rather than simply tested by them [26]. User experience is defined as “an iterative set of decisions leading to a successful outcome with an interactive tool, as well as a productive and satisfying process to achieve that outcome” [27]. To enhance the user experience of older adults when using a travel planning e-tool, it is important to design an interface that is specifically adapted to their specific needs and allows them to perform their intended tasks easily, intuitively, and without confusion or interruption [28]. Given the notable lack of studies specifically focused on developing and evaluating transport planning interfaces tailored to the needs and preferences of older adults through a participatory design approach [28], addressing this gap is essential for enhancing their user experience with transportation planning e-tools.

### Context of Study and Objective

The aim of this study is to improve older adults’ experience in using a transportation planning e-tool—called Mobilaînés—by co-designing its interface and evaluating its usability and user experience based on their needs and preferences. Rather than introducing a new mode of transportation, Mobilaînés [29] was designed to integrate existing options, helping older adults make informed travel decisions and supporting their autonomy in everyday life, using a living laboratory methodology [30]. The study took place in Sherbrooke, a city in the province of Quebec, Canada, characterized by its hilly terrain, and its main public transportation is an urban bus service. These environmental characteristics may create mobility challenges for older adults, especially those with reduced endurance, mobility limitations, or unfamiliarity with public transit. In addition, despite increasing digital technology use among older adults in Canada, digital literacy, confidence, and access remain uneven, particularly among those with functional or social vulnerabilities [31]. This context highlights the need for transportation planning tools adapted to older adults’ capabilities and preferences. The Mobilaînés living laboratory methodology, as well as its algorithm development, is described elsewhere (see Provencher et al [29] and Tahir et al [32] for additional details). The study is guided by the following research question: How does the co-designed Mobilaînés transportation planning interface improve usability and user experience for older adults?

## Methods

### Study Design

Using a participatory design approach [29], the study had 2 phases using qualitative and quantitative methods: the development phase (May 2020 to July 2022), which focused on the co-design process to ensure that older adults’ needs and preferences were considered, and the evaluation phase (August 2022 to August 2024), which examined the usability and user experience of the interface prototype to ensure alignment of the e-tool with the expectations of older adult users (Figure 1).

**Figure 1.**
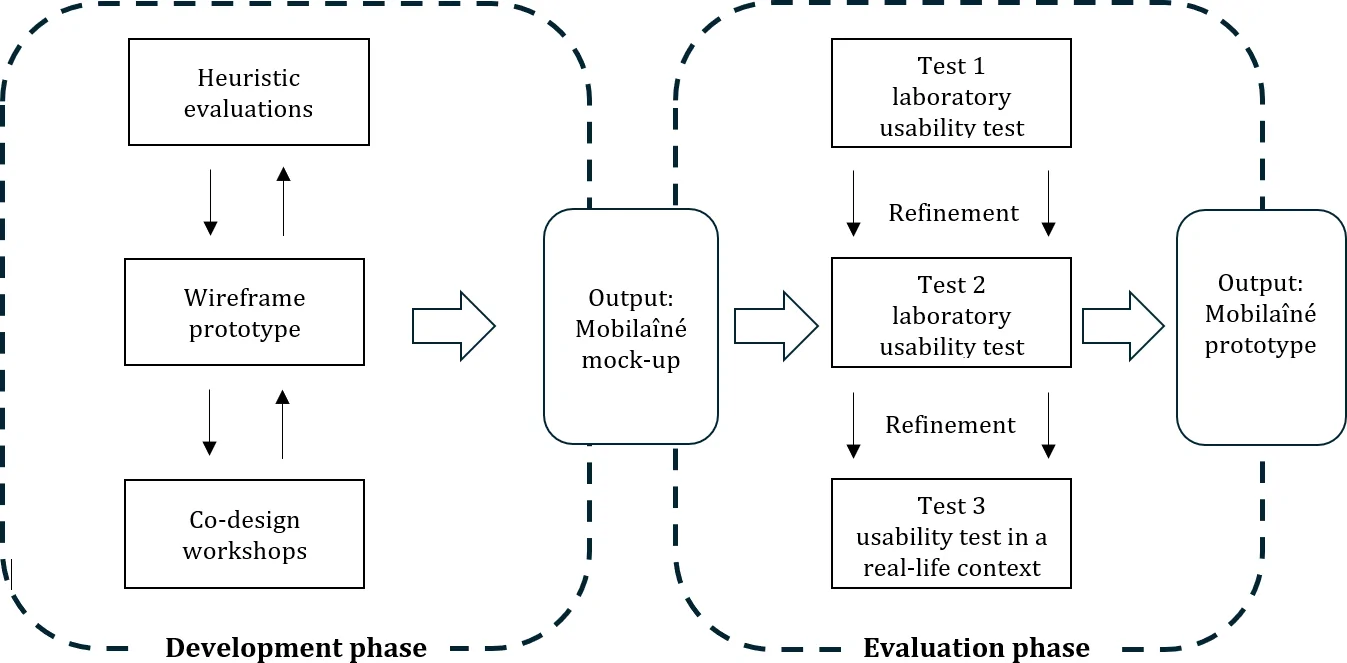
The development and evaluation process of the Mobilaînés e-tool.

### Development Phase

#### Overview

To co-design the interface of a transportation planning e-tool for older adults, we conducted a heuristic evaluation of existing transportation planning e-tools and the Mobilaînés mock-up, and we carried out a total of 17 co-design workshops with (1) the steering committee, including representatives from municipal and senior advocacy organizations (n=8); (2) the research team, comprising members from communication, computer science, and gerontology (n=10); and (3) older adults (n=5-10).

#### Heuristic Evaluation

Heuristic evaluation is a widely used expert-based method in which evaluators inspect an interface against a set of usability heuristics to identify design flaws [33], such as supporting *undo* and *redo*. We used the framework of Kumar et al [34], which includes the following phases:

##### Planning

Transportation planning e-tools that met core functional requirements, such as time autonomy (eg, customizable departure times) and weather integration, were selected in collaboration with the research team [35]. Three websites and 3 mobile apps (Android) were included: the Vermeille App and Website (the transportation planning e-tool provided by Sherbrooke Public Transit at the time of the study), the Google Maps [36] App and Website, the Moovit [37] App, and the OC Transpo [38] Website. The goal was to support the co-design of the Mobilaînés e-tool by documenting the strengths and weaknesses of these transport planning platforms and assessing their alignment with the age-friendly usability design principles and guidelines identified through a scoping review [39]. For additional details on the guidelines, see Multimedia Appendix 1.

##### Conducting

The heuristic evaluation was initially conducted by an expert evaluator (SBG), who reviewed each e-tool against the age-friendly usability design principles and guidelines identified in the scoping review. Each platform was used repeatedly and reviewed twice to explore its features, functionalities, and interaction pathways. Screenshots of each interface were captured and used during the heuristic evaluation. Findings were documented in structured notes by mapping interface elements to the corresponding heuristics, recording each guideline as compatible or noncompatible, and documenting each issue’s location, related heuristic, and potential impact on older adults’ ability to understand, navigate, or complete the task. The results were then reviewed and discussed within the research team. Identified usability issues were presented and justified using the heuristic framework, allowing collective reflection and validation of findings and helping refine the interpretation of heuristic violations and compatibility across tools.

##### Reporting

The identified compatibility and noncompatibility with heuristics were categorized and ranked by severity (minor, moderate, and major). Compatible features were summarized in a table highlighting each tool’s strengths, while noncompatible features were presented in a separate table listing usability weaknesses. The evaluation was conducted on both desktop (websites) and mobile (Android) platforms. Strengths and weaknesses of each e-tool were documented, with particular attention paid to compatibility with age-friendly principles. Recommendations for improvement were then proposed.

Based on the findings, 3 mock-ups for the Mobilaînés website were produced, reviewed with the research team and older adults during co-design activities. Their feedback informed the selection and refinement of the mock-up that best aligned with older adults’ needs and preferences, user experience considerations, and the travel-planning algorithm [28,35]. The selected mock-up was then evaluated heuristically by SBG using the same framework [34], with a specific focus on age-friendly usability principles to ensure alignment and identify areas for improvement. The evaluator reviewed the guidelines and assessed whether they were consistently applied across all dimensions of the e-tool.

### Co-Design Workshops

#### Workshops Overview

The development of the Mobilaînés e-tool was grounded in cocreation principles, particularly through workshops that brought together multiple stakeholders (eg, older adults, researchers, municipal representatives, and senior advocacy organizations) using a participatory design [28]. The methods used in the Mobilaînés co-design workshops were informed by Sanders’ [40] participatory design framework, and focused on engaging participants in generating, discussing, and refining design ideas. This approach supports the creation of concrete and actionable outputs, such as mock-ups, to inform subsequent development phases [41]. The cocreation workshops involved (1) with the steering committee to develop older adults’ personas, (2) with the research team to identify the functionalities of the Mobilaînés mock-up, and (3) with older adults. Lived experience contributors, including older adults and community representatives, played an advisory and co-design role throughout this phase. They contributed by sharing mobility experiences, reviewing personas and mock-ups, identifying usability barriers, and helping prioritize design needs. Their contributions informed the development and refinement of the Mobilaînés interface.

#### With the Steering Committee to Develop Older Adult Personas

The steering committee guided persona development using the Persona Lifecycle framework [42]. Preliminary personas were developed from semistructured interviews with 6 older adults [29] and a review of scientific and gray literature on aging, mobility, and digital inclusion. Two coconstruction workshops with stakeholders, researchers, municipal representatives, and senior advocacy organizations defined 2 dimensions: mobility profile and planning profile. Mobility profile reflected travel frequency and physical, cognitive, and financial ability to move around, resulting in 4 categories: Mobile Active, Limited Active, Limited Isolated, and Mobile Isolated. Planning profile reflected autonomy in trip planning and access to planning tools, including digital literacy and internet access, resulting in 4 categories: Autonomous Full-Access, Autonomous Limited Access, Dependent Limited Access, and Dependent Full Access. These profiles captured diverse transportation and trip-planning needs and guided recruitment, prototype development, and usage scenarios.

#### With Research Team to Identify the Functionalities of the Mobilaînés Mock-Up

The scenario-based design technique [43] was used to illustrate common travel-planning issues faced by older adults. This approach incorporated personas and “what if” reasoning to explore both the positive and negative consequences of design choices, helping to assess not only current successes but also the risks of potential changes [43]. In this regard, 3 brainstorming sessions with project team members were conducted using the scenario-based usability engineering technique [44] to (1) identify key functionalities of the e-tool, such as (i) displaying different routes, (ii) providing information on transport options, (iii) presenting travel details (eg, weather, nearby benches, and toilets), and (iv) adapting trip planning to user profiles (eg, walker users); and (2) validating the feasibility of the scenarios. In addition, 9 cocreation workshops were conducted to collaboratively design a preliminary interface for the Mobilaînés e-tool with support from an external agency. Each session involved 8-12 participants, including members of the interdisciplinary Mobilaînés team (from computer science, communication, and gerontology) and an older adult who was a member of the steering committee. Finally, insights from both the co-design sessions and the heuristic evaluation were presented to the research team, informing specific improvements to the selected mock-up ahead of the first usability test.

#### With Older Adults

Three in-person cocreation workshops were conducted with older adults, with 6-12 participants per session. Eligible participants were older than 65 years, lived in Sherbrooke, and used a computer at least weekly; those unable to travel without human assistance were excluded. Recruitment occurred through municipal and project partners, seniors’ residences, posters, and online platforms. Participants were contacted by email and phone to explain the study and assess eligibility and then selected to reflect previously developed personas varying in age, gender, mobility profile, digital competence, travel frequency, and e-tool use. The workshops documented older adults’ needs and preferences regarding the Mobilaînés interface and age-friendly features, such as customizable departure times. Feedback on the selected mock-up was documented, thematically analyzed by a research assistant, and validated by 2 principal investigators (VP and DB). Results were integrated into co-design sessions with the research team and design agency to discuss travel-planning challenges and improve the mock-up. The scenarios and mock-up were later evaluated twice by older adults to assess alignment with their expectations. Adjustments were made based on feedback, and perceptions of the interface and functionality were systematically recorded.

All workshops followed a structured but flexible format adapted to session objectives. Steering committee and research team workshops generally lasted 1-2 hours, whereas older adult workshops lasted approximately 3 hours. Sessions typically included (1) presentation of objectives and materials, such as personas, scenarios, or mock-up screens; (2) guided discussion of transportation planning needs, usability barriers, and expectations; (3) collaborative activities, such as brainstorming, scenario-based discussion, persona review, mock-up walkthroughs, or prioritization of design issues; and (4) synthesis of key decisions and next steps. Outputs included refined personas, prioritized functionalities, user scenarios, annotated mock-ups, usability issues, and design recommendations communicated to the design, programming, and research teams.

### Evaluation Phase

#### Overview

The evaluation comprised 3 experiments: 2 laboratory usability tests and 1 in-field test. According to Nielsen [33], a test with 5 users can reveal 80% of usability issues. Faulkner [45] found that groups of 10 users uncovered 95% of usability issues. Considering possible dropouts, the target sample size was 10, with a minimum of 5 to still capture approximately 80% of practical issues. The same inclusion and exclusion criteria as the co-design workshops were applied, and new participants were recruited for each test to minimize bias. A pretest was conducted with an older adult (aged 65+ years) to ensure that all test materials (eg, scenario tasks, questionnaires, and interview guide) were clear and easy to understand.

#### Usability Evaluation in a Laboratory Setting

##### Overview of Usability Evaluation

First, participants (n=7 in test 1; n=5 in test 2) tested the Mobilaînés prototype by completing 3 scenario-based tasks designed to evaluate its usability. These tasks required navigating the interface and entering travel preferences (eg, requesting assistance at the destination). Following guidance on developing usability scenarios and tasks [46], the tasks were designed to reflect realistic travel-planning activities, specify what participants were expected to accomplish, and avoid unnecessary cues about how to use the interface:

1. Task 1: planning a trip while customizing preferences to become familiar with the platform.
2. Task 2: creating and personalizing a user profile, such as saving favorite destinations, to explore additional features.
3. Task 3: planning a future round trip using all the features of the prototype, including both trip directions, save or print options, and personalized settings.

##### Objective Performance Metrics

The objective performance of participants was assessed using performance metrics to evaluate error tolerance, efficiency, and effectiveness. Observed errors were used to assess error tolerance, task duration measured efficiency (ie, the speed and productivity of task completion), and the number of completed tasks evaluated effectiveness (ie, the success rate of task completion aligned with user expectations). To ensure consistent testing conditions, 3 predefined levels of assistance were provided for participants needing help:

1. Level 1: identifying the problem and clarifying the task.
2. Level 2: providing a clue about the required information and where to find it.
3. Level 3: offering a partial solution by indicating where the information is located on the website.

Assistants (n=4) monitored screens and addressed inquiries according to these guidelines. For each subtask, the outcome was recorded as one of the following: (1) completed without assistance, (2) completed with level 1 assistance, (3) completed with level 2 assistance, (4) completed with level 3 assistance, or (5) not completed. A task or subtask was considered completed when the expected outcome was reached, independently or with predefined assistance. The task completion rate was calculated as completed tasks or subtasks divided by total assigned tasks or subtasks and expressed as a percentage; independent and assisted completion were reported separately. Microsoft Teams recordings of participants’ screens and audio were reviewed multiple times to document all observable errors across tasks and subtasks. Errors were summed across participants, tasks, and subtasks for each laboratory usability test. As there was no predefined maximum number of errors per participant or task, participants could make multiple errors and still complete a task independently. All observed errors were documented to capture ease of use, navigation challenges, and alignment with age-friendly usability guidelines. To account for different sample sizes, errors were reported as total counts and observed errors per participant.

##### Subjective Opinion (User Feedback)

Following task completion, participants completed the validated French version of the System Usability Scale (SUS) [47] to assess perceived usability and satisfaction (Multimedia Appendix 2). This questionnaire demonstrated good convergent construct validity with a single-item measure of user-friendliness (*r*=0.806) and high internal consistency (Cronbach α≥0.90) [48]. To evaluate actual usability, participants also completed the Unified Theory of Acceptance and Use of Technology (UTAUT) questionnaire (Multimedia Appendix 3) [49] to measure perceived performance expectancy, effort expectancy, facilitating conditions, and social influence. A customized Mobilaînés UTAUT model was developed to evaluate the effect of these constructs on Behavioral Intention and Behavioral Use (Multimedia Appendix 4), with content tailored to the project.

Subsequently, all participants took part in a focus group to reflect on their satisfaction with sensory navigation, information, and design. Focus group discussions explored participants’ overall experience with the Mobilaînés website, elements that enhanced or hindered satisfaction, points of confusion during task completion, comparisons with other planning tools, and their expectations (Multimedia Appendix 5). After analyzing and synthesizing the results of the first laboratory usability test, the e-tool was modified, and all testing procedures were repeated in a second round of usability testing to refine the prototype. The main modifications implemented between the first and second laboratory usability tests included clearer feedback and confirmation messages, improved step indicators and navigation cues, revised icons and colors to enhance visual clarity and consistency, simplified password and account creation guidance, and refined map icons and route-related information to support interpretation of travel options.

### Usability Evaluation in a Real-Life Setting

The effects of using the Mobilaînés prototype on older adults’ participation in meaningful activities in real-life contexts were explored. Participants (n=4), recruited using the same criteria as in previous usability tests, used the prototype over 6 weeks. Each completed a 1-hour individual introductory session, during which key functions, including account creation, trip planning, saving, and printing, were demonstrated and practiced. Two tutorial videos in French and English were uploaded to the Mobilaînés YouTube channel and emailed to participants to support use and personalization. In weeks 1, 3, and 5, participants were asked by phone to plan trips to 3 Sherbrooke locations; in weeks 2, 4, and 6, the user experience expert followed up by phone to encourage use and address difficulties. Participants were asked to use the tool at least once per week. In weeks 1 and 5, participants completed a paper version of the User Experience Questionnaire (UEQ; Multimedia Appendix 5) to assess satisfaction with the e-tool and changes in user experience over time [50]. The UEQ uses a 7-point Likert scale to assess 6 dimensions: Attractiveness, Perspicuity, Efficiency, Dependability, Stimulation, and Novelty [51] (Multimedia Appendix 6). It has acceptable-to-good reliability (Cronbach α=0.77‐0.92) and construct validity, with correlations between pragmatic and hedonic factors of *r*=0.42 (*P*<.001), and attractiveness correlating strongly with pragmatic (*r*=0.82) and hedonic (*r*=0.65) user experience aspects (*P*<.001). After 6 weeks, participants were invited to final interviews about their use of Mobilaînés, its support or lack of support for meaningful activities, and barriers and facilitators in travel planning and daily participation (Multimedia Appendix 7).

### Data Analysis

To address the first objective, selected e-tools (Vermeille app or website, Google Maps app or website, Moovit app, and OC Transpo website) were heuristically evaluated for compatibility with age-friendly usability guidelines. Compatibility was assessed descriptively across phases using the same age-friendly usability guideline framework. Applicable guidelines were classified as compatible or noncompatible, and compatibility percentages were calculated as the number of compatible guidelines divided by total applicable guidelines, multiplied by 100. Using the same framework and calculation method allowed for descriptive comparison across phases. However, the evidence used to assess compatibility differed by phase: during development, compatibility was assessed through expert heuristic evaluation, whereas during laboratory and in-field evaluations, observed usability issues, participant feedback, and evaluation findings were mapped to the same framework. An iterative deductive thematic analysis [52] was conducted by SBG on the verbatim transcripts from the cocreation workshops with older adults, using the age-friendly usability design principles and guidelines [39].

To address the second objective, descriptive analyses of participant characteristics, performance metrics, and SUS, UTAUT, and UEQ scores were conducted in SPSS (IBM Corp). SUS scores below 60 were considered unacceptable, and scores of 90 or above were considered excellent [48]. For UTAUT, a score of 3 on the 5-point Likert scale was treated as the acceptable reference value for each construct item. Focus groups and interviews were recorded, transcribed, imported into NVivo (version 20; Lumivero), and analyzed by SBG using constant comparison, deductively for usability tests and inductively for the in-field test, through open, axial, and selective coding [53]. Given the small sample sizes in the laboratory usability tests and in-field test, all quantitative findings were interpreted descriptively, and no inferential comparisons were conducted between tests. Mean and SD values were retained for questionnaire scores to remain consistent with common reporting practices for the SUS, UTAUT, and UEQ; however, these values were interpreted cautiously.

### Ethical Considerations

Ethical approval was obtained from the CIUSSS de l’Estrie-CHUS (2020‐3389: MOBILAÎNÉS). All participants provided written informed consent after being informed about the study aims and procedures. To protect privacy and confidentiality, data were coded and deidentified during collection, analysis, and reporting. Consent forms and the code key were kept securely in a locked location by the researcher in charge. Research data, including audio and video recordings, were stored confidentially in a locked location for 5 years by the principal investigator (VP). Participants in co-design workshops and laboratory usability tests received US $15 per session, with parking reimbursement when applicable; in-field test participants received US $75 each.

## Results

### Development Phase

#### Heuristic Evaluation

A comparison of 3 websites (Vermeille, Google Maps, and OC Transpo) showed that Google Maps had the strongest adherence to age-friendly usability design guidelines (see Multimedia Appendix 8 for details). The heuristic evaluation of the Mobilaînés mock-up showed 67% compatibility with age-friendly usability guidelines. While most information-related guidelines were followed, inconsistencies were noted in key principles such as Simplicity, Control, and Feedback. Specific issues included poor prioritization, repetitive actions, an excessive number of options per page, insufficient color contrast, a small font size, the presence of scroll bars, and a lack of step-by-step guidance. Only 1 guideline related to the Ease-of-Use principle (target size) was not met.

#### Co-Design Workshops

##### With the Steering Committee to Develop Older Adults Persona

Four personas were created to reflect older adults’ diverse mobility needs, abilities, contexts, and levels of digital literacy (Figure 2). Solange, a 72-year-old woman, is independent, physically active, and confident using digital tools for trip planning, representing users with high mobility and digital confidence. Léonard, a 65-year-old man with diabetes-related functional limitations, has reduced driving ability and limited digital literacy despite computer access. René, an 80-year-old man newly using a tablet provided by his family, depends mostly on others for outings but is motivated to gain independence, representing users with limited experience but willingness to learn. Suzanna, a 78-year-old recent immigrant with limited French proficiency, no social support, financial constraints, pain, and unfamiliarity with public transport, represents users with intersecting linguistic, physical, economic, and digital vulnerabilities. Solange, Léonard, and René were most relevant to Mobilaînés because they could leave home and plan trips with access to technological support. René was specifically used during heuristic evaluations to assess usability for users facing greater challenges. Suzanna could not be represented in the study because she lacked access to digital tools.

**Figure 2.**
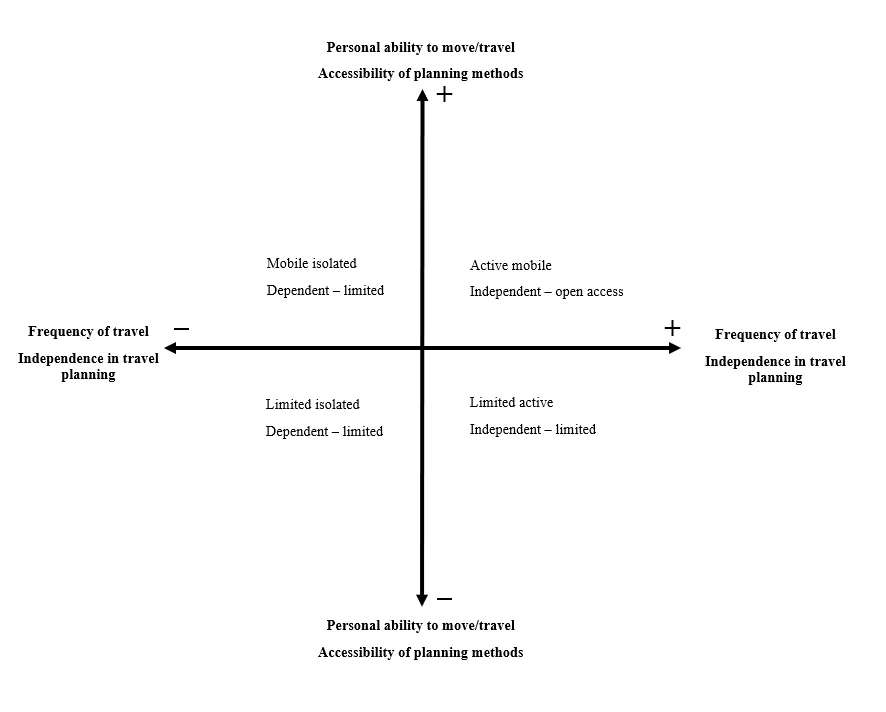
Development of personas based on older adults’ mobility and trip-planning profiles.

##### With Research Teams to Identify the Functionalities of the Mobilaînés Mock-Up

The scenario-based design technique guided the definition of the Mobilaînés mock-up features. Based on personas and older adults’ co-design feedback, the tool incorporated sheltered bus stops, restrooms, benches, low-light conditions, round-trip and future-trip planning, walking-time adjustment, hill avoidance, profile creation, favorite addresses, and travel history. To improve accessibility and reduce memory load, travel planning was divided into single-task steps, with clear information and feedback.

##### With Older Adults

Thematic analysis of older adults’ co-design feedback highlighted key usability considerations, including personalized travel planning through user profiles and simple, consistent icons, colors, and layouts. Visual clarity was a recurring concern, including font size, color contrast, and distinguishing clickable elements from informational text, such as the Mobilaînés icon and website name. Participants emphasized reducing unnecessary steps in travel planning and profile creation, improving navigation through forward or back options and map zoom, and adding text labels for unclear icons, such as the pencil icon for editing. They also noted that too many route options increased memory load and preferred highlighting 1 selected route in color. Other challenges included understanding path details and using the travel preference personalization page, which had excessive text and small icons. Finally, participants stressed the need for clear feedback after completing a plan and suggested adding a round-trip option at the start.

##### Synthesis of the Results: Heuristic Evaluation and Co-Design Workshops

Workshop themes from older adults were integrated with mock-up heuristic evaluation results and refined during a Mobilaînés project meeting. Usability issues, including color combinations, font-size buttons, and page layout, were reviewed using older adults’ feedback and age-friendly usability guidelines. SBG documented improvement ideas, and final decisions were based on feasibility and communicated to the interface design, programming, and algorithm teams to prepare the prototype for laboratory usability testing. Some preferences, such as avoiding icy routes, could not be implemented because of limited data quality and development time.

### Evaluation

#### Laboratory Usability Tests

##### Sociodemographic Overview

In the first usability test, 10 participants were recruited, but 3 were absent due to health reasons, resulting in 7 participants: 5 corresponding to Solange’s persona and 2 to Léonard’s. Participants were aged between 65 and 78 (mean 71.86, SD 4.85; median 70) years. Four participants identified as female, and 6 held a driver’s license. In the second usability test, out of 10 recruited participants, 5 were absent on the testing day, also due to health issues, resulting in 5 participants, all related to Solange’s persona. Participants were aged between 66 and 75 (mean 69.4, SD 3.36; median 69) years. In the in-field test, 4 participants aged 67-75 (mean 69.5, SD 3.79; median 68) years were recruited (see Table 1 for more details). Participants were identified using anonymized codes, where “P” refers to the participant number and “T” indicates the test session.

**Table 1.** Sociodemographic information of participants in usability tests.

Participant	Age (years)	Sex	Driving license	Means of transport	Social contact^a^	Education^b^	Revenue^c^	Health problems	Walking difficulty
P1 (T1)	78	Female	No	Bus, walking	2	2	2	Yes	No
P2 (T1)	69	Female	Yes	Car, walking	1	3	2	No	No
P3 (T1)	70	Female	Yes	Car, walking	1	2	2	Yes	No
P4 (T1)	75	Male	Yes	Car, bus	1	2	2	No	No
P5 (T1)	77	Female	Yes	Car, walking	2	2	3	No	No
P6 (T1)	69	Male	Yes	Bus, walking	1	1	2	No	No
P7 (T1)	65	Male	Yes	Bus, walking	2	1	1	Yes	Yes
P1 (T2)	66	Male	No	Adapted transport	1	1	4	No	Yes
P2 (T2)	69	Female	No	Bus, walking, taxi	1	1	5	No	No
P3 (T2)	68	Male	Yes	Car	1	1	2	No	Yes
P4 (T2)	75	Female	Yes	Adapted, walking	2	1	5	No	No
P5 (T2)	69	Male	Yes	Car, bus, walking	1	3	2	No	No
P1 (T3)	69	Female	Yes	Car, walking	1	3	2	No	No
P2 (T3)	67	Male	No	Bus, bicycle	1	3	1	No	No
P3 (T3)	67	Male	Yes	Car, car sharing	2	1	3	No	No
P4 (T3)	75	Female	Yes	Car, car sharing	1	2	2	No	No
Mean	Mean: 70.5 SD: 4.5 Median: 69	Female: 50% (N=8) Male: 50% (N=8)	Yes: 75% (N=12) No: 25% (N=4)	Car: 26.7% Walking: 30% Bus: 23.3% Adapted transport: 6.7% Taxi: 3.3% Bicycle: 3.3% Car sharing: 6.7%	1: 68.8% (N=11) 2: 31.2% (N=5)	1: 35% (N=7) 2: 31% (N=5) 3: 25% (N=4)	1: 12.5% (N=2) 2: 56% (N=9) 3: 12.5% (N=2) 4: 6.5% (N=1) 5: 12.5% (N=2)	Yes: 19% (N=3) No: 81 % (N=13)	Yes: 19% (N=3) No: 81 % (N=13)

a 1: more than once a week, 2: at least once a month.

b 1: university, 2: college, 3: secondary.

c 1: US $7k-$14k, 2: US $15k-$29k, 3: US $30k-$44k, 4: >US $59k, 5: refused.

##### Performance Metrics in Laboratory Usability Tests

Navigation, selection, insertion, and task-understanding errors were identified in both tests and used to assess error tolerance. In total, 46 errors were recorded in test 1 (n=7) and 22 in test 2 (n=5), corresponding to 6.6 and 4.4 errors per participant, respectively (Table 2). Most errors occurred during profile creation or personalization and round-trip planning. Because sample sizes differed between laboratory usability tests, errors were reported as total observed errors and errors per participant. Task performance was reported by assistance level, using counts and percentages for tasks completed independently, completed with assistance, and not completed.

Regarding *effectiveness*, measured by participants’ ability to complete tasks with or without assistance, most participants were able to complete the simpler tasks independently, while more support was required for the more complex ones (Table 3).

**Table 2.** Observed errors in laboratory usability tests.

Error metric	Test 1 (n=7)	Test 2 (n=5)
Error in task 1: 1-way trip	5	6
Errors in task 2: profile creation or personalization	18	5
Errors in task 3: round-trip planning	23	11
Total observed errors	46	22
Observed errors per participant	6.6	4.4

**Table 3.** Task completion by assistance level.

Task and subtask	Test 1 (n=7)	Test 2 (n=5)
Task 1: plan a 1-way trip	CI^a^: 4 (57%) CA^b^: 3 (43%) NC^c^: 0 (0%)	CI: 2 (40%) CA: 2 (40%) NC: 1 (20%)
Task 2a: create profile	CI: 5 (71%) CA: 1 (14%) NC: 1 (14%)	CI: 3 (60%) CA: 2 (40%) NC: 0 (0%)
Task 2b: personalize profile	CI: 0 (0%) CA: 4 (57%) NC: 3 (43%)	CI: 3 (60%) CA: 2 (40%) NC: 0 (0%)
Task 3a: plan a trip	CI: 2 (29%) CA: 3 (43%) NC: 2 (29%)	CI: 4 (80%) CA: 1 (20%) NC: 0 (0%)
Task 3b: use personalized profile information	CI: 5 (71%) CA: 0 (0%) NC: 2 (29%)	CI: 5 (100%) CA: 0 (0%) NC: 0 (0%)
Task 3c: plan a return trip	CI: 3 (43%) CA: 3 (43%) NC: 1 (14%)	CI: 3 (60%) CA: 2 (40%) NC: 0 (0%)
Task 3d: save or print	CI: 5 (71%) CA: 1 (14%) NC: 1 (14%)	CI: 4 (80%) CA: 1 (20%) NC: 0 (0%)

a CI: completed independently.

b CA: completed with assistance.

c NC: not completed.

*Efficiency* (task duration) remained relatively consistent between the 2 tests, with a mean of 7 minutes and 48 seconds for T1 and 8 minutes and 35 seconds for T2. Overall, test 2 descriptively suggested fewer observed errors per participant and higher task completion, with similar efficiency; however, these findings should be interpreted cautiously, given the small and nonequivalent participant groups.

##### Questionnaires

The mean SUS score rose from 54.6 (SD 21.2; median 57.5) to 81.5 (SD 12.6; median 82.5) between the first and second tests, shifting the interface rating from “unacceptable” to “very good” [48]. Overall, the SUS results descriptively suggest higher perceived usability after the design revisions, suggesting that these revisions improved the user experience. Mean scores of UTAUT were descriptively higher across all constructs in test 2 (n=5) than in test 1 (n=7), with Social Influence remaining the lowest-rated construct in both tests (Table 4).

**Table 4.** UTAUT^a^ scores from the first and second laboratory usability tests.

Test	Performance expectancy	Effort expectancy	Facilitating conditions	Behavioral intention	Behavioral use	Social influence
Test 1, mean (SD)	3.9 (0.7)	3.6 (1.2)	3.6 (0.8)	3.1 (1.4)	3.9 (1.4)	1.9 (1.0)
Test 2, mean (SD)	4.6 (1.1)	4.5 (1.4)	4.3 (0.6)	4.1 (0.8)	4.3 (0.7)	3.0 (1.1)

a UTAUT: Unified Theory of Acceptance and Use of Technology.

##### Focus Group Discussion

Following the 2 laboratory usability tests, participants shared valuable feedback during group discussions, which was thematically organized according to age-friendly usability design principles: Ease of Use, Structure and Navigation, Feedback, Control, Information, Simplicity, Visual Clarity, Consistency, and Accessibility (Table 5). Participants’ quotes from the laboratory usability tests were translated from French into English.

**Table 5.** Themes identified from the focus groups of the first and second usability tests.

Themes and subthemes (tests 1 and 2)	Examples of quotations
Ease of use	
Lack of automatic data entry (T1)	“I spent half the time having the right password then the email, it didn’t work. I started again, I started again, I started again” [P6, T1].
Easy to use (T2)	“I thought it [Mobilaînés] was very good because there wasn’t too much information; you had to go into it” [P5, T2].
Structure and navigation	
Difficulty in navigation (T1 and T2)	“While I was engaged, I often felt lost on the pages, unsure of how to proceed. I knew what I wanted to do, but I couldn’t figure out where to do it” [P2, T1].
Lack of using drop-down menu (T1)	“The drop-down menu was missing; when I searched for Sherbrooke, the result stayed behind the loading wheel. Usually, on the Internet, there is a small menu” [P3, T1].
Lack of visual or textual assistance (T1)	“I actually wish there were arrows” [P6, T1].
Feedback	
Lack of confirmation (T1 and T2)	“It was not easy to know when you finished the task” [P1, T1].
Lack of relevant feedback in error (T1 and T2)	“The little circle that turned, turned, turned, then gave nothing. Meanwhile, the minutes pass. I actually panicked a little” [P1, T1].
Confirmation in performing an action (T1 and T2)	“It is certain that if you use the Sherbrooke transportation system website [...] it does not ask us to confirm the new one. If our direction is correct, we know it’s [new address] correct [...]” [P1, T2].
Control	
Lack of control by undoing, redoing, and saving (T1 and T2)	“Once I had one error in the go trip or round trip, I had to go back to the option at the top [...] it took me a while to figure out” [P7, T1].
The possibility of enlarging the maps (T1)	“I was happy to see there were maps. I love that. But I haven’t tried to see if we can bring it closer because sometimes, I like to know where the stops [bus stations] are. I like to zoom in a little to see a little" [P6, T1].
Lack of safety in protecting the personal information (T2)	“Personally, I don’t go into profiles there, I’m always afraid of having my identity stolen [...] There is no recall [to change the password] [...]” [P4, T2].
Information	
Insufficient relevant information for address searches, age-related travel discounts, purchasing travel tickets, checking route conditions, and comparing different modes of transport (T1 and T2)	“I wouldn’t have known about the discount pass without my sister; it would help to integrate practical information like that” [P7, T1].
Lack of real-time travel information (T1 and T2)	“The advantage of Transit is that it provides real-time updates [...] That’s the challenge I often face: the lack of real-time awareness” [P7, T1].
Simplicity	
Lack of clarity in terminology (T1)	“About the words used when we talk about Arrive/departure, I didn’t know if it meant starting when I was performing the tasks. I was like that the more I progressed” [P3, T1].
Clarity in terminology (T1)	“About the words arrive/departure, I like it, because the departure from or arrival for that is important too” [P6, T1].
Visual clarity	
Lack of accompanying the text with images for better perception (T1)	“There are programs where when you approach the menu like personal information a small explanatory window opens” [P7, T1].
Readable font size (T1)	“Is this the real size of what will appear? Let’s say I’m on a computer. Do the writings stay big like that? I think it [font size] is okay” [P3, T1].
Lack of visual clarity in the size of text and icon (T2)	“The little instruction sign was very small” [P4, T2].
Lack of clarity in color contrast (T2)	“You need to put such type of character [color of text] or such color of character on a dark background” [P1, T2].
Consistency	
Lack of using familiar buttons, icons, or information (T1 and T2)	“I always wanted to press ‘Enter,’ but there wasn’t there [...]” [P4, T1].
Lack of presenting the information in a familiar framework and structure (T1)	“It was the American 2 am system. In Quebec, we use 14h for the afternoon” [P4, T2].
Inconsistency in navigation structure (T2)	“Maybe it would be better to put a next button instead of it [the buttons at the end of the page]” [P5, T2].
Accessibility	
Possibility of planning a trip for the future (T1)	“If you want to plan a trip for tomorrow afternoon, that’s not possible. All it gives you is leave now” [P1, T1].
Lack of adaptation to the preferences of different groups of users (T2)	“Then it [Mobilaînés] didn’t want me to take the bus, it forced me to walk. I think that’s ageism” [P5, T2].
Adaptation to the preferences of different user groups (T2)	“Totally, I should say there is a word in English, I think it [Mobilaînés] is user-friendly” [P3, T2].
Consistency with other transport planning e-tools (T2)	“I found that it was an application that worked in much the same way as other websites. The fact that we weren’t in a completely unknown universe is an advantage of it [Mobilaînés]” [P4, T2].
Possibility of personalizing the account (T2)	“Transit you can’t create an account, you always have to fill in what you want, same for Google Maps” [P5, T2].
Accessible and age-friendly transport planning website (T2)	“Totally, the Mobilaînés was good, it was pleasant. There was always something of a discovery there. But it wasn’t brilliant, but overall, it was pleasant” [P4, T2].

Across both laboratory sessions, participants encountered similar usability issues, mainly related to navigation, feedback, and data entry. Error analysis informed several refinements before test 2, including clearer confirmation messages and feedback (number 1 in Figure 3); clearer step indicators (number 2 in Figure 3); simplified password guidance (number 3 in Figure 3); renamed tabs, larger navigation arrows, improved map icons, and route controls (number 4 in Figure 3); and refinements to account creation and personalization features. Although real-time updates could not be implemented because of budget constraints, these revisions improved usability and prepared the interface for in-field testing.

In the laboratory usability evaluations, Mobilaînés’ compatibility with age-friendly design guidelines increased descriptively from 72% to 84%. Remaining issues involved color contrast, scroll bars, lack of autofill, limited feedback and control features, unfamiliar presentation of new information, and missing real-time travel updates.

**Figure 3.**
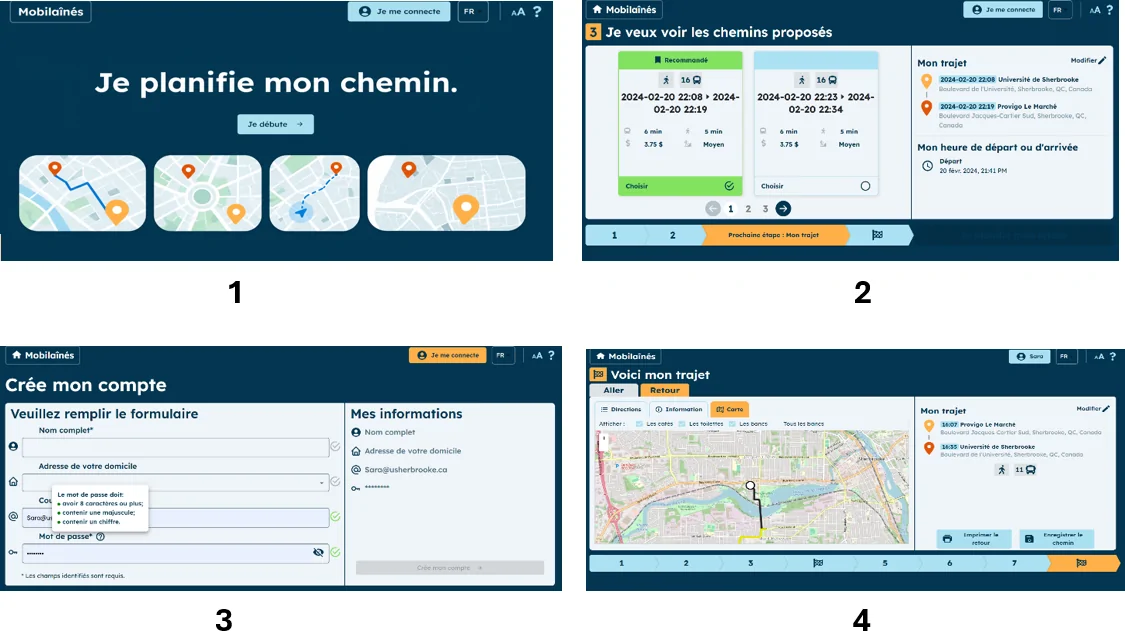
Mobilaînés website interface used for the second usability test.

### In-Field Test

#### Overview of the in-Field Test

Over 6 weeks, participants used Mobilaînés for daily trip planning. During week 1, meeting dates and locations were arranged in Sherbrooke, with 1 adjustment for a participant with limited transit experience. Two participants matching the Léonard persona required targeted support due to technical difficulties, including unfamiliarity with Google Chrome and forgetting how to use the tool. Attractiveness, Perspicuity, Efficiency, and Dependability scores were higher in the second UEQ assessment, while Stimulation and Novelty were slightly higher in the first assessment (Table 6).

**Table 6.** Scale means and SDs of the first and second UEQ^a^ assessments.

UEQ scale	First UEQ, mean (SD)	Second UEQ, mean (SD)
Attractiveness	1.46 (0.91)	2.08 (1.13)
Perspicuity	0.88 (0.48)	1.69 (1.13)
Efficiency	1.50 (0.54)	1.69 (1.09)
Dependability	0.81 (0.90)	2.13 (0.85)
Stimulation	1.69 (1.07)	1.38 (1.18)
Novelty	0.94 (1.30)	0.81 (1.84)

a UEQ: User Experience Questionnaire.

Comparison of the UEQ scores with the standardized benchmark dataset revealed that Stimulation consistently ranked in the top 25% (good) across both assessments, while Novelty remained in the top 50% (above average). Efficiency and Attractiveness scores increased from the top 50% (above average) to the top 25% (good) and top 10% (excellent), respectively. Dependability and Perspicuity showed the greatest improvement, moving from the bottom 50% (below average) to the top 10% (excellent) and top 50% (above average), respectively (Figure 4).

**Figure 4.**
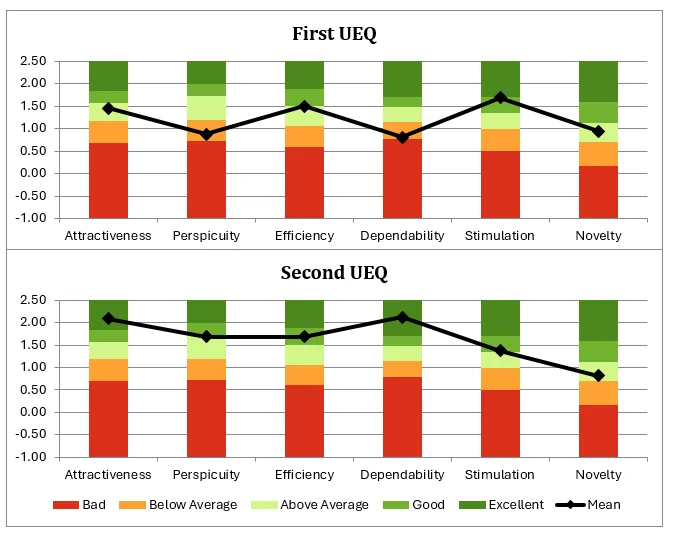
UEQ benchmark comparison for the Mobilaînés e-tool: first vs second assessment. UEQ: User Experience Questionnaire.

#### Interviews

A total of 14 subthemes were identified and classified into 9 key themes (Table 7). Five of these themes reflected positive content, including ease of use, an age-friendly travel-planning process, tailored and optimal route options, accurate timing and information, and reliability.

**Table 7.** Themes and subthemes identified through the in-field test interview.

Themes and subthemes	Examples of quotations
Ease of use	
Easy to navigate and functional e-tool for travel planning Easy-to-use systematic travel-planning process	“I find that although Mobilaînés has more steps, each window focuses me on a specific question [...] so I do give Mobilaînés more stars [...]” [P3].
Absence of real-time and traffic information	
Lack of real-time travel and traffic information	“[...] for planning, I would use Mobilaînés. For the actual travel time, I would give the advantage to Google Maps because it provides real-time bus stop information, and I would know where I am [*...*] if Mobilaînés had real-time information, I would still use Mobilaînés first for planning [*...*]” [P3].
Age-friendly travel-planning process	
Easy and accessible possibility of planning a round-trip Detailed information provided on the path, which navigates users in the routing process.	“I used to use Google Maps, but [*...*] I prefer Mobilaînés because it provides more of what I expect [*...*]” [P2].
Tailored and optimal route options	
Route options more tailored to user needs and preferences Different route options in travel planning	“[*...*] anytime I’ve compared Mobilaînés to Google Maps for the proposed bus routes, they’ve always been the same, or Mobilaînés has been at least as good, if not better. [*...*] So, it was the best options were provided by Mobilaînés. [*...*] I would look at Mobilaînés and for going to new locations” [P3].
Accurate timing and information	
Accurate and precise travel-related information Accurate timing in travel planning	“It encouraged me to go out more. [*...*] The mobile app would show me exactly what time to take the bus to Carrefour. [*...*] So, you could say that the timing information on the website is one of the motivators [*...*]” [P2].
Reliability	
Mobilaînés is reliable for going to new places Mobilaînés is reliable for going out independently	“The first time I was very, very apprehensive [*...*] But now no problem. Now I feel comfortable, and I trust it” [P4].
Lack of accuracy in navigation	
Mobilaînés is not that reliable in walking as it sometimes shows the wrong path	“[*...*] that was the main bug for me [*...*] it was always having me go in the wrong direction or not in the proper route [*...*]” [P3].
Absence of pertinent feedback	
Lack of feedback in displaying the error message	“When you put the time around noon, forget it. [...] At one time, I could not print. [...] It’s okay that that [printing error] happened only once” [P4].
Necessity to minimize walking time	
Lack of possibility of saving the walking time	“[*...*] For me, it’s not a problem, but for an older person [*...*] it will take a long time to get to the bus. [*...*] for people who are not used to walking, it’s difficult [*...*]” [P1].

In sum, the in-field usability evaluation suggested that the iterative co-design and testing process led to an interface largely aligned with age-friendly guidelines, reaching 95% compatibility. Key design decisions included maintaining a step-by-step travel-planning process, presenting structured route information, supporting personalization of travel preferences, and improving visual cues and feedback. These design changes appeared to support participants’ confidence and perceived reliability of the e-tool in real-world travel planning. Remaining issues included inconsistent presentation of new information, limited simplification of some travel data, lack of real-time travel and traffic information, and technical problems such as poor error handling and incorrect time displays. These issues were forwarded to the development team for correction.

## Discussion

### Principal Findings

This study sought to enhance the experience of older adults using a transportation planning e-tool by developing an interface grounded in their needs and preferences through a participatory approach. The process focused on 2 objectives: co-designing an interface with older adults and evaluating its usability and user experience. The compatibility of the Mobilaînés e-tool with age-friendly design guidelines increased descriptively from 67% to 95% through iterative mock-up development and usability tests. These improvements were reflected in user experience scores, moving from unacceptable to very good.

The compatibility of the Mobilaînés e-tool with age-friendly heuristics aligns with findings by Patsoule and Koutsabasis [54], who identified Visibility and Ease of Understanding as the 2 primary heuristics tailored to older adults in website redesign. A clear and intuitive interface can significantly reduce cognitive load for older adults when interacting with digital tools, making tasks more manageable and less stressful [55]. Our results are also in line with those of Hart et al [47], who suggested that improvements to increase design compatibility with guidelines contributed to a more user-friendly experience for older adults. In our study, in-field test participants expressed a high level of satisfaction with the website’s interface, which was easy to use and well suited to their travel planning needs. UEQ results showed that after several weeks of use, participants found the Mobilaînés e-tool more interesting, practical, and easy to use for travel planning. With repeated use, they also perceived it as more attractive, efficient, and trustworthy, encouraging more spontaneous and sustained engagement. However, one participant noted the absence of real-time travel and traffic information, which would have allowed tracking of their bus journey and arrival times. This limitation was observed across all 3 usability evaluations and relates to the *Information* principle in usability design guidelines. Although the travel plan printing feature does not offer real-time updates, its visual and informative format ensures accessibility for all older adults, regardless of digital proficiency. For older adults who may experience heightened anxiety from unexpected travel disruptions, Citroen [56] emphasizes that access to up-to-date information can significantly reduce stress and improve decision-making. Incorporating such features not only enhances the tool’s practicality but also strengthens user confidence and satisfaction—key factors in promoting adoption and sustained use.

Interpreted through the UTAUT model [49], the design refinements implemented in Mobilaînés may help explain improvements in usability and acceptance. Step-by-step navigation, simplified task flow, familiar terminology, and clearer feedback may have supported effort expectancy by reducing the perceived difficulty of use. Personalized route-planning options, such as saved addresses and walking preferences, may have strengthened performance expectancy by making the tool more useful for users’ everyday travel needs. Clear structured guidance may also represent facilitating conditions by helping users complete tasks with greater confidence. From a behavior change perspective [57], these features may support perceived capability and confidence in planning trips independently, which may encourage future use and engagement in community mobility.

However, achieving these improvements required several design and evaluation cycles, highlighting the time and resources needed to develop age-friendly e-tools through participatory and iterative approaches [58]. Qualitative data further highlighted the tool’s reliability, accessibility, and effectiveness for transportation planning, despite limitations such as the absence of real-time data. Overall, these findings emphasize the value of involving older adults throughout the design and evaluation process to ensure that the Mobilaînés e-tool is closely attuned to their lived experiences and everyday contexts [58,59].

The in-field test results further revealed that Mobilaînés reduced participants’ travel-related concerns by helping them anticipate each step of their journey and boosting their confidence in reaching unfamiliar destinations or adopting new modes of transport, particularly when transitioning away from car use. Although the destinations and usage period of the Mobilaînés tool were predetermined, participants reported that it enhanced trip predictability and served as a reliable resource for leaving their homes independently. In line with behavior change theory [57] and the Technology Acceptance Model [60], generating positive emotions, such as feeling safe and anticipating the pleasure of reaching a destination, may encourage sustained use of Mobilaînés. Greater ease of use enhances the overall experience, which can in turn support older adults’ independence, social participation, and overall quality of life [61]. According to Schroeder et al [62], older adults’ intention to adopt technology is notably influenced by awareness, emotional needs, and motivational aspects. Understanding these motivators is essential, as personal desire drives activities such as visiting family, whereas medical appointments are often perceived as obligatory [63]. This finding highlights the potential of Mobilaînés to not only facilitate independent mobility but also foster sustained engagement in meaningful daily activities by aligning technological support with older adults’ emotional and motivational needs.

The main contribution of this study is not limited to the development of a transportation planning e-tool; rather, it lies in generating practical design lessons for age-friendly transportation planning interfaces. Through an iterative co-design and evaluation process, this study identifies concrete design decisions related to navigation, feedback, visual clarity, personalization, and route information that can inform the development of future digital transportation tools for older adults.

### Limitations

This study faced several limitations. First, recruitment challenges resulted in an overrepresentation of tech-savvy participants (Solange personas) and an underrepresentation of those with lower digital literacy (Leonard and René personas). This imbalance may have influenced usability outcomes and should be considered when interpreting the findings. The use of personas also requires caution, as they can simplify the diversity of older adults’ experiences if they are not continuously grounded in lived experience. In this study, personas were developed based on interviews, literature, and stakeholder workshops, and were used as design tools to guide recruitment, scenario development, and interface decisions. However, the overrepresentation of participants corresponding to the Solange persona may have influenced design priorities, limiting the representation of other mobility and digital literacy profiles. Additionally, the small sample size in the laboratory usability tests (n=5 and 7) and particularly in the in-field test (n=4) warrants caution regarding the generalizability and transferability of the findings. The in-field results should therefore be interpreted as exploratory, providing preliminary insight into real-world use rather than definitive evidence of sustained effectiveness. Moreover, comparisons between the 2 laboratory usability tests should be interpreted descriptively, as they involved different participants and participant profiles, with the second test including only those participants corresponding to the Solange persona. Therefore, observed improvements in performance metrics, SUS, UTAUT, and UEQ scores may reflect both interface refinements and differences in participant characteristics, rather than generalizable evidence of effectiveness. Several strategies were used to promote recruitment, such as online advertisements, poster distribution, and the use of local databases. However, given the risks of fraud associated with soliciting older adults by phone or online [64], recruitment through trusted individuals or community partners may be particularly important in future studies [65]. Moreover, flexible scheduling regarding time and place helped to reduce withdrawal due to health reasons, which is common in participatory approaches involving older adults [66,67].

Second, resource constraints affected the feasibility of conducting a broader summative evaluation, such as involving multiple independent evaluators for heuristic analysis. The iterative co-design process required more design and evaluation cycles than anticipated, creating challenges related to time, resources, and project continuity. This highlights the need to plan sufficient resources and procedural continuity from the outset, as the number of refinement cycles can be difficult to predict. To support methodological rigor, usability issues were reviewed with interdisciplinary team members during key development and evaluation phases to help reduce interpretation bias [68]. Design issues were prioritized according to their relevance to age-friendly usability principles, potential impact on task completion and user experience, and feasibility within project constraints.

From a reflexivity and positionality perspective, the analysis and design decisions were shaped by an interdisciplinary team with expertise in user experience, rehabilitation, gerontology, communication, and computer science. While this positioning supported a multidimensional interpretation of the findings, it may also have influenced how usability issues were identified and prioritized. To reduce this risk, interpretations were informed by multiple sources of evidence, including heuristic evaluation results, older adults’ feedback, performance metrics, questionnaire results, focus groups, and interviews.

### Future Studies

Future studies should address key challenges identified during the development and evaluation of the Mobilaînés application. First, integrating real-time travel and traffic data is essential for effective trip planning [69], reducing anxiety about getting lost [10], protecting against adverse weather and traffic [70,71], and supporting transport choices aligned with individual needs [72]. Features not yet implemented, such as avoiding icy roads, could be enhanced through technical solutions and partnerships, improving adaptation to real-life mobility challenges and addressing safety concerns for pedestrians using mobility devices in winter conditions [73-75].

Second, future research should examine design approaches that support adoption and long-term routinization among older adults with diverse mobility, social participation, and digital literacy profiles. Interview and focus group findings suggested that accessibility features, such as pairing icons with text labels and using familiar visual cues, may benefit all users while supporting age-related visual decline and novice users, such as those represented by the René and Suzana personas. Older adults’ technology adoption is shaped by prior experience, available support, perceived usefulness, confidence, and comfort [21]. For Mobilaînés, these factors may involve reliability, accurate real-time and offline information, auditory guidance, and cues that provide ongoing support or training. Universally accessible systems responsive to older adults’ needs can improve usability and user experience, thereby encouraging adoption and sustained engagement [76,77]. Finally, future studies should include longitudinal evaluations of spontaneous and sustained real-world use beyond structured, researcher-supported testing to assess long-term impacts on meaningful activities, gradual behavioral changes, and infrequent travel needs, such as medical appointments.

### Conclusions

Given the aging population and the critical role of mobility in health outcomes [78], this study addresses a key knowledge gap by advancing age-friendly digital transport planning. Using a participatory approach, the Mobilaînés e-tool was developed and evaluated with older adults to ensure that the interface aligned with their needs and preferences. A key contribution of this study is the identification of concrete design improvements, such as step-by-step task flow, clearer navigation, enhanced visual cues and feedback, structured route information, and personalization of travel preferences that support a more age-friendly planning experience. Findings highlight the need for iterative refinement to reduce cognitive load, support meaningful engagement, and promote digital inclusion, while guiding the development of accessible transport technologies for older adults. Future research should examine human-centered and technological strategies to foster adoption and sustained use, particularly among users with limited digital literacy.

## Supplementary material

10.2196/88297 Multimedia Appendix 1 Age-friendly usability design principles and guidelines (from Givi et al [39]).

10.2196/88297 Multimedia Appendix 2 System Usability Scale (SUS) questionnaire.

10.2196/88297 Multimedia Appendix 3 Unified Theory of Acceptance and Use of Technology (UTAUT) questionnaire.

10.2196/88297 Multimedia Appendix 4 Mobilaînés Unified Theory of Acceptance and Use of Technology (UTAUT) Model and definition of the core constructs.

10.2196/88297 Multimedia Appendix 5 Focus group guide for laboratory usability tests.

10.2196/88297 Multimedia Appendix 6 User Experience Questionnaire (UEQ) structure and questionnaire.

10.2196/88297 Multimedia Appendix 7 Interview guide for the in-field usability tests.

10.2196/88297 Multimedia Appendix 8 Heuristic evaluation.

## Abbreviations

SUS: System Usability Scale
UEQ: User Experience Questionnaire
UTAUT: Unified Theory of Acceptance and Use of Technology
