# Supplementary material for: Development and Evaluation of a Transportation Planning e-Tool for Older Adults: Co-Design Study
Source: JMIR Aging. 2026 Oct 23;9:e88297. doi: 10.2196/88297 (PMC13693824; doi:10.2196/88297)
Supplement: Supplementary file 1 [file aging-v9-e88297-s001.docx]

**Multimedia Appendix 1**- **Age-friendly usability design principles and guidelines (from Givi et al., submitted).**

| **Principles** | **Guidelines** |
| --- | --- |
| Feedback | - Provide informative feedback by showing clicked links, task status, waiting periods, and error messages with resolution steps. - Request confirmation before performing actions that could cause significant changes or data loss, and confirm successful actions |
| Consistency | - Maintain consistency in design elements, across pages and screens such as using familiar icons, input formats, colors, menu hierarchy, and so on. - Present information clearly by using familiar frameworks and structures, and apply consistent navigation patterns and layouts. - Underline text only when it is a link. |
| Minimizing the Memory load | - Present one task or process at a time. - Avoid complex dynamic input fields that require multiple gestures or the use of both hands. - Eliminate unnecessary or repetitive actions. - Limit the number of key functions on the home screen to 12 or fewer for tablets and 6 or fewer for smartphones. - Avoid changes in familiar behavior to speed up the actions of experienced users. - Use images, videos, or audio to complement text information. - Minimize time-sensitive content and provide options to adjust or extend time limits if necessary. |
| Simplicity | - Simplify data entry processes - Use simple and easy-to-understand graphics. - Ensure simplicity, clarity, and consistency in screen layout, navigation, and terminology. - Provide simple error messages (not codes). |
| Accessibility | - Consider user profiles in design such as their digital literacy, and visual and cognitive impairment. - Ensure button accessibility with a universal approach, accommodating left-handed users. - Use icons alongside text labels. - Include alternative data entry methods for users unable to use the primary method. - Ensure the application interface adapts to device rotation and user attempts to enlarge screen content. - Provide content in various multimedia formats, such as text, videos, pictures, and audio. |
| Structure and Navigation | - Avoid requiring users to double-click or drag to interact with the page. - Offer both click-to-zoom and two-finger zoom gestures. - Minimize data entry steps to ideally fewer than three. - Provide help tutorials or brief animated instructions. - Clearly indicate touchable or clickable elements. - Position key elements (e.g., buttons, information) at the center of the screen. - Guide users with systematic navigation to indicate their location and the next step. - Provide a virtual keyboard for numerical or alphabetical entry. - Use a short, simple, and static pull-down menu. - Include spell check for errors. - Maintain hierarchy in feedback, navigation, visual design, and data grouping. - Avoid using scroll bars, especially horizontally. - Position search tools at the top right. |
| Control | - Provide the zoom up ability up to 200%. - Consider the home menu as a safe return point. - Ability to control requests by undoing, redoing, and exiting. - Avoid disappearing messages; let users close them manually. - Offer adjustable text sizes, color schemes, and contrast to enable personalization of the user experience. |
| Ease of use | - Enable a function to remember usernames and passwords. - Make account creation optional for users. - Incorporate auto-save features whenever possible. - Provide support for both orientations. - Include automatic data entry (e.g., time, date) to reduce data entry time and keyboard use. - Ensure easy access to help and support. - Consider target touch areas of at least 9mm x 9mm, with a minimum of 8 dp of inactive space between them. |
| Visual Clarity | - Avoid using fluorescent colors and colors with minimal contrast, such as green and red, yellow and blue, or green and blue. - Avoid using all capital letters words. - Consider 3:1 and 4:1 color contrast ratio for text and large text respectively. - Adjust the contrast range between 1.7 and 3.0. - Use blue, red, and magenta as the link colors on a white background due to their high visibility. - Utilize brightness levels of 75% and 50%. - Use font sizes of 12 pt to 14 pt for body text and 16 pt for titles. - Utilize sans-serif fonts like Arial, Helvetica, and Tahoma. - Avoid justified alignment, unfilled text, text shadows, large blocks of italic or bold text, and underlined text. - Maintain line spacing between 1.5 to 2. - Consider using bold typefaces for emphasis. - Avoid complex and distracting elements such as animations, blinking text, patterned backgrounds, pure white backgrounds, pure black text on white backgrounds, and transparent menus. |
| Information | - **The website should not include pop-up messages or advertisements.** - **Avoid unclear or ambiguous language.** - **Do not use abbreviations or symbols.** - **Ensure each page has a clear and descriptive title.** - **Avoid using Roman numerals.** - **Use short sentences (less than 70 characters per line) and paragraphs (each focusing on a single idea) and avoid hyphenating words at the end of lines.** - **Highlight important information for better visibility.** - **Use active voice to enhance clarity.** - **Use names that are conceptually related to function.** |
