# Supplementary material for: Development and Evaluation of a Transportation Planning e-Tool for Older Adults: Co-Design Study
Source: JMIR Aging. 2026 Oct 23;9:e88297. doi: 10.2196/88297 (PMC13693824; doi:10.2196/88297)
Supplement: Supplementary file 2 [file aging-v9-e88297-s002.docx]

**Multimedia Appendix 2: System Usability Scale (SUS) Questionnaire**

For each statement, please indicate your level of agreement on a scale from 1 to 5.

1 = Strongly disagree; 5 = Strongly agree

If you are unsure how to respond to a statement, please select the middle point of the scale (score 3).

| **SUS items** | Strongly disagree | Disagree | Neutral | Agree | Strongly agree |
| --- | --- | --- | --- | --- | --- |
| 1. I think that I would like to use this tool frequently | 1 | 2 | 3 | 4 | 5 |
| 2. I found the tool unnecessarily complex | 1 | 2 | 3 | 4 | 5 |
| 3. I thought the tool was easy to use | 1 | 2 | 3 | 4 | 5 |
| 4. I think that I would need the support of a technical person to be able to use this tool | 1 | 2 | 3 | 4 | 5 |
| 5. I found the various functions in this tool were well integrated | 1 | 2 | 3 | 4 | 5 |
| 6. I thought there was too much inconsistency in this tool | 1 | 2 | 3 | 4 | 5 |
| 7. I would imagine that most people would learn to use this tool very quickly | 1 | 2 | 3 | 4 | 5 |
| 8. I found the tool very cumbersome to use | 1 | 2 | 3 | 4 | 5 |
| 9. I felt very confident using the tool | 1 | 2 | 3 | 4 | 5 |
| 10. I needed to learn a lot of things before I could get going with this tool | 1 | 2 | 3 | 4 | 5 |
