# Supplementary material for: Development and Evaluation of a Transportation Planning e-Tool for Older Adults: Co-Design Study
Source: JMIR Aging. 2026 Oct 23;9:e88297. doi: 10.2196/88297 (PMC13693824; doi:10.2196/88297)
Supplement: Supplementary file 3 [file aging-v9-e88297-s003.docx]

**Multimedia Appendix 3: Unified Theory of Acceptance and Use of Technology (UTAUT) Questionnaire**

For each statement, please indicate your level of agreement on a scale from 1 to 5.

1 = Strongly disagree; 5 = Strongly agree

If you are unsure how to respond to a statement, please select the middle point of the scale (score 3).

| **UTAUT items** | Strongly disagree | Disagree | Neutral | Agree | Strongly agree |
| --- | --- | --- | --- | --- | --- |
| 1. Mobilaînés is an effective tool for planning a trip. | 1 | 2 | 3 | 4 | 5 |
| 2. Mobilaînés is a useful tool for me that meets my trip-planning needs. | 1 | 2 | 3 | 4 | 5 |
| 3. Using Mobilaînés increases my productivity in planning a trip. | 1 | 2 | 3 | 4 | 5 |
| 4. I can quickly plan a trip with Mobilaînés. | 1 | 2 | 3 | 4 | 5 |
| 5. The trip-planning process in Mobilaînés is clear and easy to understand. | 1 | 2 | 3 | 4 | 5 |
| 6. I found it easy to learn how to plan a trip in Mobilaînés. | 1 | 2 | 3 | 4 | 5 |
| 7. I am more likely to use Mobilaînés if people around me (friends and family) use it. | 1 | 2 | 3 | 4 | 5 |
| 8. People around me (friends and family) think I should use a web tool like Mobilaînés to plan my trip. | 1 | 2 | 3 | 4 | 5 |
| 9. I have the necessary knowledge to plan my trip in Mobilaînés. | 1 | 2 | 3 | 4 | 5 |
| 10. I feel in control when I use Mobilaînés. | 1 | 2 | 3 | 4 | 5 |
| 11. I think using Mobilaînés fits well with the way I like to plan a trip. | 1 | 2 | 3 | 4 | 5 |
| 12. I intend to use Mobilaînés in the future. | 1 | 2 | 3 | 4 | 5 |
| 13. I am willing to recommend the use of Mobilaînés to people around me. | 1 | 2 | 3 | 4 | 5 |
| 14. It is a good idea to use Mobilaînés to plan a trip. | 1 | 2 | 3 | 4 | 5 |
| 15. I like using Mobilaînés. | 1 | 2 | 3 | 4 | 5 |
| 16. Using Mobilaînés makes trip planning more interesting for me. | 1 | 2 | 3 | 4 | 5 |
