# Supplementary material for: Development and Evaluation of a Transportation Planning e-Tool for Older Adults: Co-Design Study
Source: JMIR Aging. 2026 Oct 23;9:e88297. doi: 10.2196/88297 (PMC13693824; doi:10.2196/88297)
Supplement: Supplementary file 4 [file aging-v9-e88297-s004.docx]

**Multimedia Appendix 4: Mobilaînés UTAUT Model and definition of the core constructs**

| **Constructs** | **Definition (Venkatesh, et al., 2003)** | **Adapted the definition to Mobilaînés** |
| --- | --- | --- |
| Performance Expectancy (PE) | “The degree to which an individual believes that using the system will help him or her to attain gains in job performance.” | The degree to which older adults perceive using Mobilaînés improves their travel planning performance |
| Effort Expectancy (EE) | “The degree of ease associated with the use of the system” | The degree of ease associated with the use of Mobilaînés |
| Social Influence (SI) | “The degree to which an individual perceives that important others believe he or she should use the new system.” | The degree to which older adults perceive that the acceptance of using Mobilaînés is influenced by the people around them. |
| Facilitating Conditions (FC) | “The degree to which an individual believes that an organizational and technical infrastructure exists to support the use of the system” | The degree to which older adults believe that an organizational and technical infrastructure exists to support the use of Mobilaînés. |
| Behavioral Intention (BI) | “An individual's overall affective reaction to using a system.” | The degree to which older adults perceive their intention to use Mobilaînés |
