# Supplementary material for: Development and Evaluation of a Transportation Planning e-Tool for Older Adults: Co-Design Study
Source: JMIR Aging. 2026 Oct 23;9:e88297. doi: 10.2196/88297 (PMC13693824; doi:10.2196/88297)
Supplement: Supplementary file 5 [file aging-v9-e88297-s005.docx]

**Multimedia Appendix 5: Focus group guide for laboratory usability tests**

1. What was your overall experience in planning a trip with Mobilaînés?
2. What made you satisfied or dissatisfied with this experience? (For example, colors, icons, readability, easy/difficult navigation on the site, etc.)
3. Different features (text, icons, color, site navigation, etc.) are offered on the different pages of Mobilaînés. Which features guided you well in planning your trip? And which features hindered you in planning your trip? Why?
4. When you encountered an error during use, did you know how to correct it? If yes, what led you to correct it?
5. Have you ever used electronic trip planning tools? Is Mobilaînés different? If yes, how is it different?
6. Is there anything else you would like to mention regarding the use of Mobilaînés? For example, if you could change something in Mobilaînés, what would it be? What would be your suggestions?
