# Supplementary material for: Development and Evaluation of a Transportation Planning e-Tool for Older Adults: Co-Design Study
Source: JMIR Aging. 2026 Oct 23;9:e88297. doi: 10.2196/88297 (PMC13693824; doi:10.2196/88297)
Supplement: Supplementary file 6 [file aging-v9-e88297-s006.docx]

**Multimedia Appendix 6: User Experience Questionnaire (UEQ) structure and questionnaire**

For each statement, you are asked to position yourself on a scale of 1 to 7 regarding your use of the Mobilaînés website.

1= Completely disagree; 7 = Completely agree

If you do not know how to position yourself in relation to a statement, you are invited to respond anyway by checking the center of the scale (score 4)

| Annoying | 1 | 2 | 3 | 4 | 5 | 6 | 7 | Enjoyable |
| --- | --- | --- | --- | --- | --- | --- | --- | --- |
| Not Understandable | 1 | 2 | 3 | 4 | 5 | 6 | 7 | Understandable |
| Creative | 1 | 2 | 3 | 4 | 5 | 6 | 7 | Dull |
| Easy To Learn | 1 | 2 | 3 | 4 | 5 | 6 | 7 | Difficult To Learn |
| Valuable | 1 | 2 | 3 | 4 | 5 | 6 | 7 | Inferior |
| Boring | 1 | 2 | 3 | 4 | 5 | 6 | 7 | Exciting |
| Not Interesting | 1 | 2 | 3 | 4 | 5 | 6 | 7 | Interesting |
| Unpredictable | 1 | 2 | 3 | 4 | 5 | 6 | 7 | Predictable |
| Fast | 1 | 2 | 3 | 4 | 5 | 6 | 7 | Slow |
| Inventive | 1 | 2 | 3 | 4 | 5 | 6 | 7 | Conventional |
| Obstructive | 1 | 2 | 3 | 4 | 5 | 6 | 7 | Supportive |
| Good | 1 | 2 | 3 | 4 | 5 | 6 | 7 | Bad |
| Complicated | 1 | 2 | 3 | 4 | 5 | 6 | 7 | Easy |
| Unlikable | 1 | 2 | 3 | 4 | 5 | 6 | 7 | Pleasing |
| Usual | 1 | 2 | 3 | 4 | 5 | 6 | 7 | Leading Edge |
| Unpleasant | 1 | 2 | 3 | 4 | 5 | 6 | 7 | Pleasant |
| Secure | 1 | 2 | 3 | 4 | 5 | 6 | 7 | Not Secure |
| Motivating | 1 | 2 | 3 | 4 | 5 | 6 | 7 | Demotivating |
| Meets Expectations | 1 | 2 | 3 | 4 | 5 | 6 | 7 | Does Not Meet Expectations |
| Inefficient | 1 | 2 | 3 | 4 | 5 | 6 | 7 | Efficient |
| Clear | 1 | 2 | 3 | 4 | 5 | 6 | 7 | Confusing |
| Impractical | 1 | 2 | 3 | 4 | 5 | 6 | 7 | Practical |
| Organized | 1 | 2 | 3 | 4 | 5 | 6 | 7 | Cluttered |
| Attractive | 1 | 2 | 3 | 4 | 5 | 6 | 7 | Unattractive |
| Friendly | 1 | 2 | 3 | 4 | 5 | 6 | 7 | Unfriendly |
| Conservative | 1 | 2 | 3 | 4 | 5 | 6 | 7 | Innovative |
