# Supplementary material for: Development and Evaluation of a Transportation Planning e-Tool for Older Adults: Co-Design Study
Source: JMIR Aging. 2026 Oct 23;9:e88297. doi: 10.2196/88297 (PMC13693824; doi:10.2196/88297)
Supplement: Supplementary file 7 [file aging-v9-e88297-s007.docx]

**Multimedia Appendix 7: Interview guide for the in-field usability tests**

1. Can you describe your experience since the last time you used Mobilaînés?? For example, were you able to plan your trip without hassle? Choosing the best transport option? Arrive at your destination on time? etc.
2. Can you mention three elements that make it easier to use Mobilaînés to carry out your outdoor activities?

Outdoor activities: For example, visiting a family member or friend, going to a medical appointment, ...

Elements that make use easier: for example, it is easy to use, the information provided is sufficient or useful, etc.

3. Can you mention three elements that make it more difficult to use Mobilaînés to carry out your outdoor activities?

Outdoor activities: For example, visiting a family member or friend, going to a medical appointment, ...

Elements that make it difficult to use: for example, it is difficult to use, the information provided is insufficient or useless, etc., it is difficult to plan a journey.

4. Were you comfortable going to new places with Mobilaînés? Please explain…
5. Has Mobilaînés encouraged you to leave your home more frequently? How? Please explain…
6. If you had to choose between Mobilaînés and the travel planning app or website (for example google maps or Transit) you use the most, which would you choose? Why?

**Appendix 8: Heuristic evaluation**

Heuristic evaluation of the selected tools.

Compatibility of each e-tool based on the age-friendly usability design guidelines.
