# Supplementary material for: Development and Evaluation of a Transportation Planning e-Tool for Older Adults: Co-Design Study
Source: JMIR Aging. 2026 Oct 23;9:e88297. doi: 10.2196/88297 (PMC13693824; doi:10.2196/88297)
Supplement: Supplementary file 8 [file aging-v9-e88297-s008.docx]

**Multimedia Appendix 8: Heuristic evaluation**

Heuristic evaluation of the selected tools.

Compatibility of each e-tool based on the age-friendly usability design guidelines.
